# Acute Polio-Encephalitis and Polio-Myelitis

**Published:** 1905-07-29

**Authors:** 


### Hospital Clinics.

### ACUTE POLIO-ENCEPHALITIS AND POLIO-MYELITIS.

Dr. Leonard Guthrie [1] gives an interesting account of the acute cerebral paralyses of children which depend upon an acute encephalitis. He points out that it is now recognised that infantile paralysis is the same disease whether the lesion affects, as it most commonly does, the motor cells in the anterior cornua of the spinal cord, or the motor cells in any part of the upper motor tract. The evidence in favour of this unity of the disease is largely circumstantial. It is well known that infantile paralysis occurs epidemically, and in such epidemics one member of a family may be stricken with a cerebral lesion while another may show the typical symptoms of frank anterior polio-myelitis. Thus, Dr. William Pasteur has recorded the case of a family of nine children, of whom five were seized almost simultaneously with a febrile illness. One of these, a boy of 12 years, developed on the third day a paralysis of the ordinary spinal form affecting the whole of the left upper limb. A second boy, aged nine years, developed a spastic hemiplegia on the right side on the seventh day of his illness, with transient paralysis of the right side of the face and palate. This was obviously a cerebral lesion. A third child on the fifth day showed a rigid paralysis of the left lower limb, with wasting and loss of knee-jerk in the affected member. This was probably a spinal lesion. In two others tremors lasting a few days followed the fever, and in the case of one of them a transient strabismus was noted. Here, again, the lesion was probably cerebral.

The morbid anatomy of acute anterior polio-myelitis gives the picture of an acute congestion accompanied by thrombosis of the smaller vessels in the affected area, with round-celled exudation, and small hæmorrhages in the grey matter of the anterior horns supplied by the anterior spinal arteries. The secondary results of the lesion are anæmic softening of the areas involved, and ultimately absorption of the necrotic products and cicatrisation. In the case of the acute cerebral palsies the morbid picture has been shown by Dr. F. E. Batten to be precisely identical with the above, and the pathological unity of the two diseases is placed beyond doubt. Of the ætiology little definite is known. The appearance of the two varieties of the disease in epidemic form, and their special prevalence during certain months of the year, notably the late summer and early autumn, point to an acute infection by micro-organisms, but so far no specific microbe has been associated with the lesion. None the less the balance of probability is in favour of a specific infection, although a good number of cerebral cases have been reported as occurring in the wake of other acute specific fevers such as measles, whooping cough, scarlet fever, diphtheria, and influenza.

The invasion of both varieties is marked by constitutional symptoms of variable gravity. In the spinal form the invasion is sudden and associated with fever, vomiting, pains in the head, back, and limbs. In a few hours or days one limb or more may be found flaccid and motionless. In the early stages the affected limbs are acutely tender to the touch, but in time all the symptoms subside, leaving an entire limb, or certain groups of muscles in it, in a state of flaccid paralysis. The muscles waste, the tendon reflexes disappear, the temperature of the limb falls below that of its fellow, and the electrical reaction of degeneration is established in the paralytic muscles. The paralysis is always more extensive at first than it is likely to prove ultimately, and the prognosis must therefore be guarded by a tendency to optimism.

In the cerebral form the invasion is similar, but, as might be expected, the symptoms are more severe and lasting; in addition to the symptoms enumerated for the invasion of the spinal form there may be delirium, or stupor, or convulsions. As is the case with the spinal variety the ultimate lesions are seldom as severe as the early stages of the malady suggest.

Acute encephalitis has been divided for purposes of clinical description into two forms, a superior and an inferior, according as the lesion is situate in the upper or lower brain areas.

1. Polio-encephalitis superior may affect:

*a.* The præfrontal convolution, in which case profound and lasting mental changes may be expected.

*b.* The motor areas either in the cortex or in the descending motor tract, giving rise to hemiplegia or diplegia.

*c.* The cerebellum or its peduncles, in which case the result is a disturbance of equilibrium and ataxia.

*d.* The occipital lobes, where it will probably occasion blindness due to involvement of the double half-vision centres.

2. Polio-encephalitis inferior is the name applied to lesions of the disease situate below the corpora quadrigemina. In such cases the result is ophthalmoplegia of different kinds, or, when the bulbar nuclei are attacked, bulbar palsy of a degree varying with the extent of the involvement.

The author has collected a series of illustrative cases, an outline of some of which will assist the conception of acute polio-encephalitis in varying situations.

Case 1. A child, previously healthy and in all respects normal, was attacked, at the age of two-and-a-quarter years, with a series of convulsions, followed by a stupor which lasted for a week. At the end of the week, on recovering consciousness, she was unable to talk. She could stand, but could not walk, although there was no loss of muscular power, nor any alteration of the normal reflexes. She was an idiot of a most degraded type, and showed no sign of improvement for the six months during which she was kept under observation. Dr. Guthrie considers that the lesion was certainly a præfrontal one.

Case 2. A girl of six years, who, after an onset somewhat similar to the last, gradually passed into a condition of typical cerebellar ataxy. She has gradually improved, and, though lethargic, is not mentally deficient. The lesion is considered to have been cerebellar.

Case 3. A boy of twelve who, a month after a mild febrile attack of a nature doubtful at the time, was gradually attacked by a right hemiparesis, of cerebral type, associated with loss of vision in the opposite eye. He recovered his strength completely, but his vision in the affected eye remained impaired. Dr. Guthrie considers that the symptoms depended upon a thrombosis of terminal branches of the left Sylvian artery, and of its offshoots to the left optic nerve—*i e.* a retro-bulbar neuritis.

Case 6a. *Encephalitis of Occipital Lobes.*—The case of a child who became completely blind for three weeks without any other sign of ocular disease. Sight was gradually regained, but the patient became subject to *petit mal.*

Case 7. *Acute Bulbar Palsy.*—A boy of nine had a sudden febrile attack, on the second day of which he choked while taking his medicine, could not laugh or cough, and could not speak intelligibly. His temperature rose to 104° and remained high for a week. At the end of the fourth week all symptoms had subsided with the exception of slight weakness in swallowing. The knee-jerks were normal, and it is considered that a diphtheric origin for the paralysis can be excluded.

In conclusion, the author observes that a recognition of the fact that acute encephalitis is by no means a very rare disease will assist in the avoidance of confusion between it and tuberculous meningitis—a point of much importance in view of the great difference in the outlook of the two diseases. He observes also that the conception of a primary thrombosis as the underlying feature of the brain lesions, affords a simple explanation of recovery following symptoms which appear, from their extent, to depend upon most extensive involvement of brain tissues. A mild and temporary condition of thrombosis is compatible with complete restoration of function, and the degree of recovery will vary inversely with the amount of structural damage occasioned by the thrombosis. The interesting suggestion is made that many cases of paralysis generally regarded as hysterical may depend upon a capillary thrombosis, and that the fleeting paralyses which often usher in disseminated sclerosis may similarly own a thrombotic origin.

[1] Clin. Jour., July 5, 1905.

